# Prevalence, burden and risk markers of health problems in HYROX athletes: a 12-week prospective cohort study

**DOI:** 10.3389/fspor.2026.1937574

**Published:** 2026-09-09

**Authors:** Emilia Chittenden, Amy Bell, Georgia Forbes, Phoebe Tooth, Ruby Porter, Rosie Van Der Vliet, Kareem Shouman, Sean Williams

**Affiliations:** 1 Department for Health, Centre for Health and Injury and Illness Prevention in Sport, University of Bath, Bath, United Kingdom; 2 UK Collaborating Centre on Injury and Illness Prevention in Sport (UKCCIIS), Bath, United Kingdom

**Keywords:** athletic injuries, high-intensity functional training, hybrid athlete, risk marker, sports surveillance

## Abstract

**Introduction:**

HYROX is a rapidly growing fitness competition combining eight 1-km running intervals with eight functional fitness stations. This study investigated the epidemiology and markers of health problems in HYROX athletes.

**Method:**

Eighty-nine HYROX athletes (56 female) completed the ‘Oslo Sports Trauma Research Center Questionnaire on Health Problems’ (OSTRC-H2) weekly for 12 weeks, alongside exposure reporting and a baseline questionnaire. In an exploratory analysis, potential risk markers (age, sex, previous injury and HYROX experience) for cumulative injury severity were examined using negative binomial regression with an exposure offset.

**Results:**

The mean weekly response rate was 62%. The pooled weekly prevalence of any health problem was 30% (95% CI 24% to 37%), with a substantial-problem prevalence of 18% (95% CI 13%–24%). Injury incidence was 14.4 (95% CI 11.1–18.4) per 1,000 h, with a time-loss incidence of 6.4 (95% CI 4.3–9.2); injuries attributed to HYROX training accounted for two thirds of episodes (9.6 per 1,000 h). Overuse injuries accounted for 73% of injuries. The knee was the most frequently injured region (22% of episodes) and, with the lower leg, carried the greatest burden; the elbow ranked third for burden on the basis of four episodes, two of them persistent, while shoulder injuries were near-absent. Previous injury carried the largest rate ratio for cumulative severity, at 2.60 (95% CI 1.00–6.75), an interval also compatible with no association.

**Conclusions:**

Health problems were common in HYROX athletes and dominated by overuse injuries. The burden was led by the lower limb, consistent with the sport's running load, with a possible elbow contribution from two persistent injuries and a near-absence of shoulder problems, a combination that may distinguish the profile from other extreme conditioning programme training but which rests on few cases. Only half of problems involved time-loss, so missed-training-day surveillance understates the burden. Previous injury is a candidate risk marker requiring confirmation in larger cohorts.

## 1 Introduction

HYROX is a standardised global indoor fitness competition consisting of eight 1-km running intervals interspersed with eight functional fitness stations: 1 km SkiErg, 50 m sled push, 50 m sled pull, 80 m burpee broad jump, 1 km rowing, 200 m kettlebell farmers carry, 100 m sandbag walking lunge, and 100 wall balls (1). The format sits within the broader category of high-intensity functional training (HIFT), combining endurance running with resistance-based exercises in a fixed, repeatable structure. Since its launch in Germany in 2017, HYROX has expanded rapidly, with participation projected to exceed one million competitors during the 2025/26 season (2, 3). This growth has outpaced the evidence base. Only one study has examined injury occurrence in HYROX, in which 44% of respondents answered affirmatively to a single retrospective questionnaire item asking whether they had sustained any injury related to HYROX training (4). However, no recall window was specified, so the period over which respondents reported varied with how long each had been training, leaving the estimate without a common time base across the sample and compounding the recall bias inherent to retrospective designs (5). Furthermore, no information was recorded on symptom severity, functional consequence or time loss, nor on training exposure, so neither burden nor incidence can be derived and the figure cannot be placed against any other population. As such, no exposure-adjusted estimate is currently available of how frequently HYROX athletes sustain health problems, which structures are affected, or the severity of those problems.

**Table 1:** Baseline participant characteristics of HYROX athletes (*n* = total analysed).

Characteristic	*N* = 89
Age group	
16–29	25 (28%)
30–39	24 (27%)
40–49	24 (27%)
50+	16 (18%)
HYROX experience	
0–6 months	25 (28%)
6 months—1 year	30 (34%)
1–2 years	15 (17%)
2–3 years	8 (9%)
3 + years	6 (7%)
Sex: female	56 (63%)
Total training, h/wk	7.0 (6.0–10.0)
HYROX-specific training, h/wk	3.0 (2.0–4.0)
Total exposure, h	37.5 (13.1–70.1)
Weeks responded	9.0 (3.0–12.0)
Previous injury (time-loss) in last 12 months	39 (44%)
Competed in last 12 months	41 (46%)

Values are n (%) for categorical variables or median (IQR) for continuous variables. Percentages for HYROX experience do not sum to 100% due to missing responses (n = 5).

In the absence of HYROX-specific data, injury risk can only be inferred from the modalities that HYROX combines. A meta-analysis of running-related injury reported a weighted incidence of 7.7 per 1,000 h in recreational runners and 17.8 per 1,000 h in novice runners, with individual study estimates spanning 2.5–33.0 and heterogeneity in injury definition identified as a principal limitation on comparison across studies (6). A systematic review of resistance training modalities reported incidence rates spanning 0.21–18.9 per 1,000 h, averaging 4.2 per 1,000 h across seventeen studies of high-intensity functional training, 4.0 per 1,000 h in powerlifting and 3.2 per 1,000 h in weightlifting; no incidence estimate was recoverable for traditional strength training, and the authors attributed the near ninetyfold spread across studies principally to the absence of a standardised injury definition, seven of the included studies having defined injury not at all (7). In extreme conditioning programme training specifically, an incidence of 3.2 per 1,000 h has been reported under a definition requiring at least three days of time-loss or medical attention (8). Variation across these estimates is therefore driven as much by the injury definition and the exposure denominator as by the activity being measured, and the figures cannot be combined to predict a rate for an activity that draws on all three. Direct measurement, with both the injury definition and the denominator stated, is required before HYROX can be positioned against any of them.

There are also structural grounds for expecting that the HYROX profile will not simply interpolate between its constituent modalities. Approximately 60% of race duration is spent running, which makes the format running-dominant, but the running is not performed continuously: eight 1-km intervals alternate with eight stations, so every run after the first is performed in a progressively fatigued state and every station is entered carrying the residual fatigue of the run preceding it (9, 10). The stations demand high force output under substantial metabolic stress, and several of them, among them the sled push, sled pull, farmers carry and sandbag lunge, load the body in patterns that neither distance running nor conventional resistance training rehearses at comparable intensity. The sequence is also fixed. Unlike many extreme conditioning programmes, in which the daily stimulus is varied by design, every HYROX race comprises the same eight movements in the same order, so preparation converges on a narrow and heavily repeated movement set. Training that combines substantial running volume with high-volume repetition of a small number of loaded patterns would be expected to generate gradual-onset problems, and to distribute them differently from either running or gym-based resistance training. Whether it does so has not been established.

Prospective injury surveillance requires instruments sensitive enough to capture the full spectrum of health problems. Traditional time-loss definitions miss a substantial proportion of conditions that affect training and competition, particularly overuse injuries (11, 12). The Oslo Sports Trauma Research Centre Questionnaire on Health Problems (OSTRC-H2) was developed to address this limitation, recording symptoms and functional consequences regardless of time-loss, and in a direct comparison detected around three times as many injuries as a time-loss definition applied to the same athletes (13). It has not yet been applied in HYROX, a format in which repeated running is combined with high-volume loaded functional stations, and where a considerable burden of overuse problems is plausible but would go largely undetected by time-loss surveillance methods.

The primary aim of this study was to describe the weekly prevalence, incidence, anatomical distribution, and burden of health problems among HYROX athletes over a 12-week period. A secondary, exploratory aim was to identify potential risk markers for cumulative injury severity, to generate hypotheses for future confirmatory research.

## 2 Materials and methods

### 2.1 Study design and participants

A 12-week prospective cohort study was conducted between 1 December 2025 and 1 March 2026. Participants were recruited via emails to HYROX-affiliated gyms, flyer distribution across the University of Bath campus, and social media. Prize draws (3 × £25, 2 × £50, 1 × £100 Amazon vouchers) were conducted every four weeks to promote participation, contingent on 80% questionnaire completion.

Eligible participants were aged 16 years or older, fluent in English, participating in HYROX-specific training at least once per week, with at least three weeks of HYROX training experience. Participants with a current medical condition likely to substantially alter training were excluded. Electronic informed consent and baseline demographics were obtained via QuestionPro. Ethical approval was granted by the University of Bath Research Ethics Committee (REF 12792-15539), and all procedures were conducted in accordance with the Declaration of Helsinki.

No *a priori* power analysis was performed; sample size was governed by resource and time constraints, a legitimate and reportable basis where scope is externally bounded (14). As an undergraduate dissertation project limited to a 12-week semester window and without funds for participant payment or advertising, recruitment yielded 89 participants. This provided sufficient person-weeks to estimate the prevalence, incidence and burden of health problems with reasonable precision, the study's primary aim, though the secondary risk marker analysis was correspondingly underpowered and is reported as exploratory.

### 2.2 Health problem definitions

A health problem was defined as any condition representing a deviation from an individual's normal state of full health, regardless of its impact on participation, performance, or help-seeking behaviour (15). Problems were classified as acute injuries, overuse injuries, or illnesses. All injuries were included in the primary injury-specific analyses, regardless of the activity to which they were attributed; injuries that participants attributed to HYROX training, including related running and strength training, were additionally analysed as a pre-specified subset in sensitivity analyses. Injury mechanism was recorded as strength-related, running-related, or HYROX station-related. Onset was classified as new, recurrent, or chronic/worsening.

### 2.3 Data collection

Participants completed the OSTRC-H2 weekly, distributed each Sunday with a 48-h reminder to non-responders. The four questionnaire items generate a severity score from 0 (no problem) to 100 (maximum burden). Following Clarsen, Bahr (15), a health problem was classified as “substantial” if it caused a moderate or severe reduction in training volume (item 2) or in performance (item 3), or complete inability to participate; on the standard item scoring this corresponds to a score of 17 or above on either item. “Time-loss” problems were those involving at least one missed training day. By this definition all time-loss problems are also substantial. All injuries were reported *de novo* each week, with repeated reports of chronic conditions consolidated into single unique injury events. Weekly exposure was reported separately for running, resistance training, HYROX conditioning, recovery work (prehabilitation and stretching), and competitions. Total exposure for each participant was the sum of these five components across all weeks in which a questionnaire was returned, so exposure accrued only over observed weeks. Recovery work accounted for 12.5% of total reported hours; as prehabilitation and stretching are not exposure to the loading patterns that likely generate the injuries recorded here, incidence was additionally calculated with recovery hours removed from the denominator (Supplementary Table S1). Weekly exposure was reported separately for running, resistance training, HYROX conditioning, recovery work (prehabilitation and stretching) and competition. Total exposure was the sum of these five components across returned weeks, so exposure accrued only over observed weeks. All incidence rates use this total as the denominator, including those for the HYROX-attributed subset, so that the primary and sensitivity estimates remain directly comparable. Recovery work accounted for 12.5% of reported hours; because prehabilitation and stretching are not exposure to the loading patterns that generate these injuries, incidence was additionally calculated with recovery hours removed (Supplementary Table S1). Injury mechanism, recorded as running, strength or station work, corresponds directly to three of the reported exposure components, and episodes with a recorded mechanism were related to the matching modality exposure as an exploratory analysis. A mechanism was recorded for only 30 of the 63 episodes, so these rates are reported separately and are not comparable with the primary estimate (Supplementary Table S2).

### 2.4 Statistical analysis

All analyses were conducted in R version 4.5.2 (R Core Team, 2025), using the MASS (16), sandwich (17), and boot (18) packages. Prevalence was computed by pooling across all returned participant-weeks, as the number of participant-weeks in which a health problem was reported divided by the total number of participant-weeks returned, separately for all health problems, overuse injuries, acute injuries and illnesses. Pooling rather than averaging the twelve weekly proportions weights each participant-week equally and so avoids giving disproportionate influence to weeks with few respondents. Point estimates and 95% CIs were obtained by cluster bootstrap with 2000 replicates, resampling participants with replacement so that the interval accounts for repeated measurement within participants. Injury episodes were reconstructed from the weekly panel using explicit rules. An episode was opened only at a week reporting a new injury onset. It was continued through subsequent injury weeks that did not report a new onset, provided that the body region was unchanged and that no intervening returned questionnaire reported the participant to be free of injury. A returned questionnaire reporting no injury, or a change of body region, closed the episode. Weeks for which no questionnaire was returned did not close an episode, so a problem reported either side of a missing week was treated as continuing rather than recurring. Cumulative severity and time-loss days were summed within each episode; as these rules are partly arbitrary where reporting is incomplete, incidence was recalculated under alternative rules in which a gap of more than two weeks, more than one week, or any missing week closed an episode, and under the opposing assumption that unattached injury weeks represented incident episodes (Supplementary Table S1). Injury incidence rates were expressed as new episodes per 1,000 h of total exposure, with exact Poisson 95% CIs, reported overall and by sex, with the HYROX-attributed subset reported as a sensitivity analysis. One injury episode, reported by a participant who provided no exposure data, was excluded from all injury analyses; this participant was retained in the baseline description and the prevalence estimates, to which they contributed one person-week. The anatomical distribution of injuries was tabulated by body region, injury type (acute vs. overuse) and time-loss category, following the IOC consensus statement classification (19). Relative burden was quantified as the product of injury frequency (injuries per 1,000 exposure hours) and mean cumulative OSTRC-H2 severity score for each body region, and illustrated using a risk matrix following Bahr, Clarsen (20). The matrix included all injuries and was restricted to the eight highest-burden body regions. Illnesses were excluded from the matrix because only reported symptoms (not formal diagnoses) were captured, but illness counts and severity distribution are reported in Table 3.

Non-response was examined in three ways. The distribution of completed weeks per participant was described, and the missingness pattern was classified as monotone where a participant's returned questionnaires formed an unbroken run from week 1. Whether non-response was related to health status was assessed by comparing the probability of returning a questionnaire in week t + 1 according to whether a health problem had been reported in week t, tested with Fisher's exact test. Baseline characteristics were compared between participants above and below the median number of completed weeks using standardised mean differences. Weekly prevalence was recalculated under two alternative assumptions: last observation carried forward across gaps lying between two returned questionnaires, without extrapolation beyond a participant's final response; and all non-responses treated as problem-free, which provides a lower bound (Supplementary Tables S3, S4). Potential risk markers for cumulative injury severity were examined using negative binomial regression. The outcome was the cumulative injury severity score summed across all injury episodes, with log (total exposure hours) as an offset, so that exponentiated coefficients are interpretable as rate ratios. The same model was refitted with the cumulative HYROX-attributed injury severity score as a sensitivity analysis. Negative binomial was preferred over Poisson to accommodate overdispersion, and HC3 heteroscedasticity-consistent robust standard errors were used throughout. Covariate selection was guided by existing sports injury epidemiology theory: previous injury with time-loss, the most consistently replicated risk marker in the sports injury literature (21); sex, reflecting established biological and sociocultural differences in injury patterns (22, 23); age, given known changes in tissue resilience and recovery capacity (24); and HYROX experience, a proxy for sport-specific conditioning and technical proficiency (25). Total exposure hours were handled via the offset term. This analysis was exploratory and intended to generate hypotheses for future confirmatory research.

## 3 Results

A total of 89 athletes were recruited. Of these, 56 (63%) were female (Table 1). Questionnaires were returned for 658 participant-weeks of a possible 1,068, a mean weekly response rate of 62%. Five participant-weeks had been submitted twice and were reduced to a single record. Completion was unevenly distributed rather than uniformly partial: 32 participants (36%) completed all 12 weeks and 45 (51%) completed nine or more, whereas 30 (34%) completed three or fewer (median 9 weeks, IQR 3–12). For 60 of 89 participants (67%) the returned questionnaires formed an unbroken run from week 1, so most missing data arose from withdrawal rather than from intermittent non-response. At baseline, 39 participants (44%) reported a previous injury with time-loss.

Non-response showed no clear relationship with health status. A questionnaire was returned in the following week after 88.4% of weeks in which no health problem had been reported, and after 83.5% of weeks in which one had (odds ratio 0.66, *p* = 0.118), a difference in the direction of injury-related withdrawal but small and imprecisely estimated. Participants completing at or above the median of nine weeks did not differ materially at baseline from those completing fewer, all standardised mean differences being 0.22 or below (Supplementary Table S3). Pooled prevalence of any health problem was 29.9% across returned participant-weeks, 29.9% when gaps between two returned questionnaires were filled by carrying the last observation forward, and 18.4% under the extreme assumption that every non-response represented a problem-free week (Supplementary Table S4). Because exposure accrued only over weeks in which a questionnaire was returned, incidence numerators and denominators were restricted to the same observed weeks; onsets occurring during unobserved weeks and subsequently reported as ongoing would have been counted as continuations rather than onsets, so the incidence rates reported here are more likely to be underestimates than overestimates.

**Figure 1:**
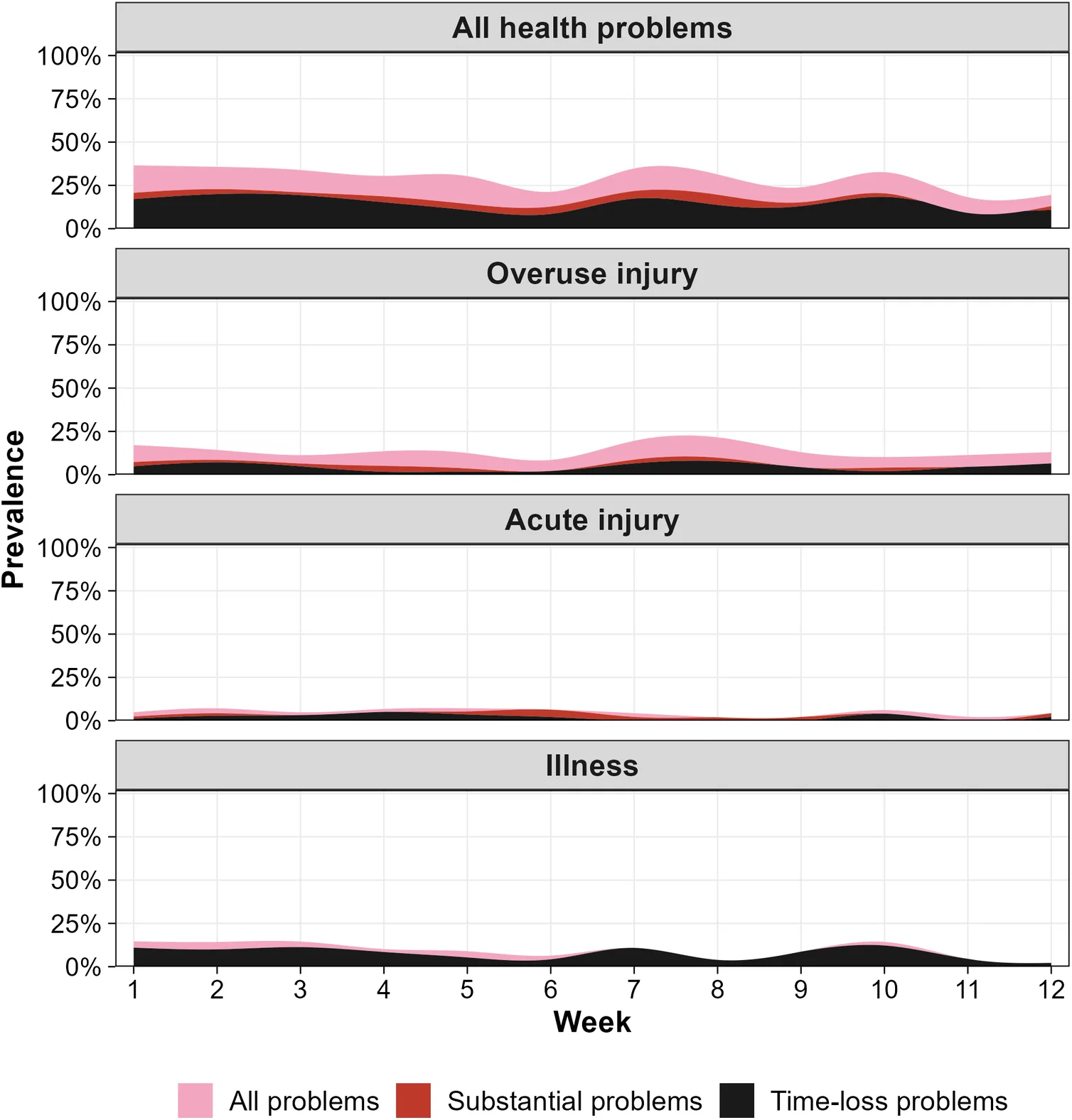
Prevalence of all health problems, overuse injuries, acute injuries and illness.

During the 12-week surveillance period, 68 of 89 participants (76%) reported at least one health problem. The pooled weekly prevalence of any health problem was 30% (95% CI 24%–37%). Substantial problems had a weekly prevalence of 18% (95% CI 13%–24%) and time-loss problems 15% (95% CI 11%–19%). Injuries that participants attributed to HYROX training accounted for a mean weekly prevalence of 13% (95% CI 8%–19%), of which 6% (95% CI 3%–10%) were substantial and 5% (95% CI 2%–8%) were time-loss problems. Overuse injuries (14%; 95% CI 9%–20%) were more prevalent than acute injuries (5%; 95% CI 2%–10%) throughout the study period. The mean weekly prevalence of illness was 10% (95% CI 7%–13%). Figure 1 shows the weekly variation in prevalence across the 12-week period.

Participants reported 63 new injury episodes over 4,376 h of total exposure, giving an overall injury incidence rate of 14.4 (95% CI 11.1–18.4) per 1,000 h (Table 2). For time-loss injuries specifically (≥1 missed training day), the incidence rate was 6.4 (95% CI 4.3–9.2) per 1,000 h. Injuries that participants attributed to HYROX training accounted for two thirds of episodes, giving a HYROX-attributed incidence of 9.6 (95% CI 6.9–13.0) per 1,000 h and a HYROX-attributed time-loss incidence of 4.3 (95% CI 2.6–6.8) per 1,000 h. Incidence was robust to the construction of the exposure denominator and to the episode-reconstruction rules (Supplementary Table S1).

**Table 2:** Injury incidence rates by sex (all injuries).

Sex	Athletes	Exposure (*h*)	Injuries (*n*)	Overall IR/1,000 h (95% CI)	Time-loss (*n*)	Time-loss IR/1,000 h (95% CI)
Male	28	1,488	26	17.5 (11.4 to 25.6)	11	7.4 (3.7 to 13.2)
Female	56	2,734	37	13.5 (9.5 to 18.7)	17	6.2 (3.6 to 10.0)
Unspecified	4	154	0	0.0 (0.0 to 24.1)	0	0.0 (0.0 to 24.1)
Combined	88	4,376	63	14.4 (11.1 to 18.4)	28	6.4 (4.3 to 9.2)

Sex was not recorded for four participants, shown as unspecified.

**Figure 2:**
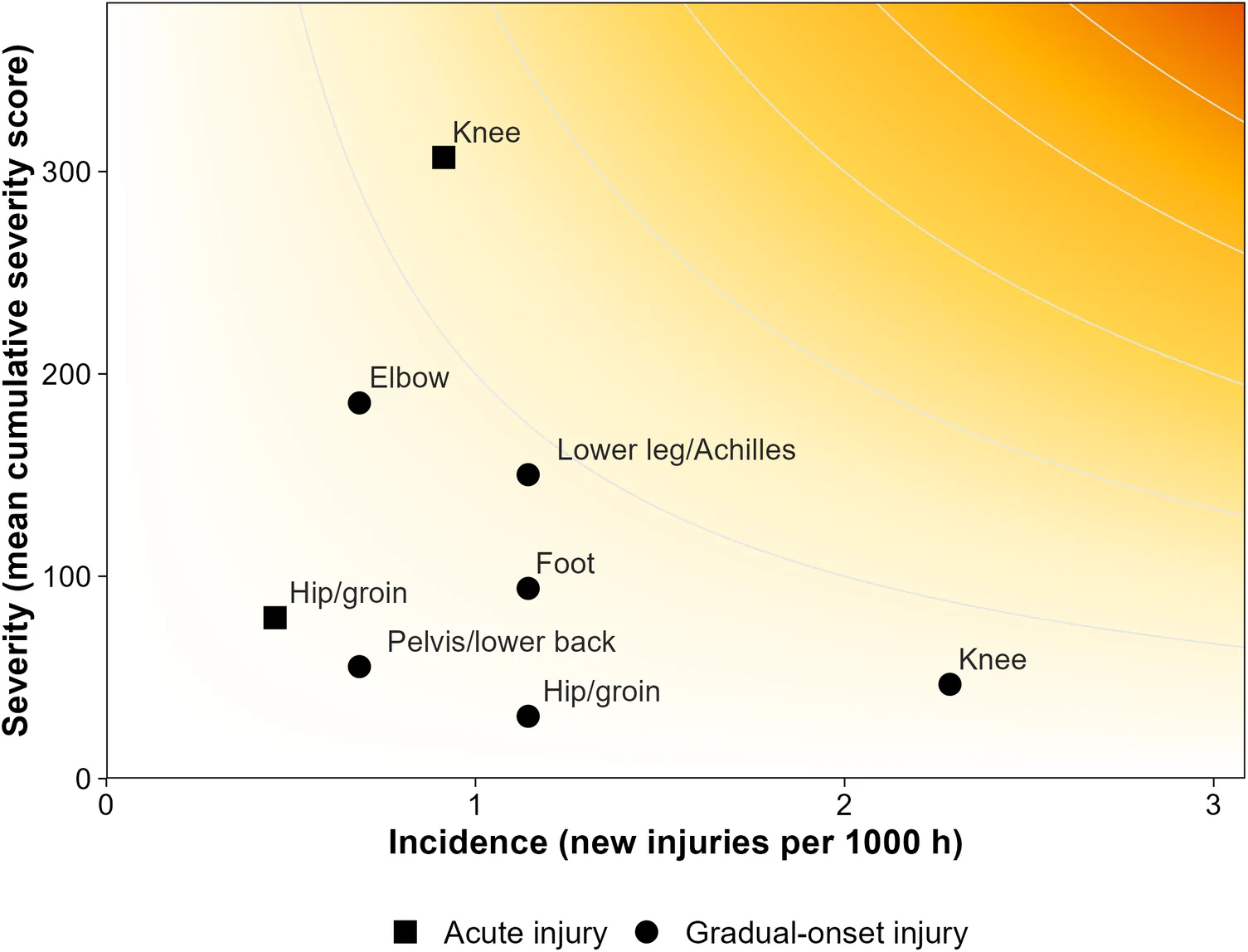
Burden matrix illustrating the incidence and mean cumulative severity scores of the eight highest-burden injury location and type combinations. Circles: gradual-onset (overuse); squares: acute. Illness is excluded (see Table 3 for illness counts).

Table 3 shows locations for acute injuries, overuse injuries and illnesses as well as time-loss. Overuse injuries at the knee, hip/groin, foot, and lower leg/Achilles were the most common injury locations, whilst illnesses accounted for 57% of all days lost. A mechanism was recorded for 30 of the 63 injury episodes (48%), and less often for injuries that participants did not attribute to HYROX training (67% missing) than for those they did (44% missing). Among the 30 episodes with a recorded mechanism, running accounted for 43%, HYROX-specific station work for 30%, and strength training for 27%. Where a mechanism was recorded, running predominated at the knee (5 of 9), the lower leg and Achilles (2 of 2) and the foot (2 of 3), whereas the three elbow episodes with a recorded mechanism were attributed to station work or strength training; mechanisms were mixed at the hip, groin, pelvis and lower back. When these counts were related to the corresponding modality-specific exposure, running carried the highest rate, at roughly twice that of strength training, although the confidence intervals overlapped substantially (Supplementary Table S2). Given the extent and the differential nature of the missing mechanism data, this comparison should be treated as exploratory. Figure 2 illustrates the relative burden of injuries by body region. The knee, lower leg/Achilles and elbow represented the greatest injury burdens; the knee and lower leg combined a high injury frequency with high average severity, whereas the elbow burden arose from a small number of persistent injuries.

**Table 3:** Injury and illness locations and time-loss in HYROX athletes.

Body region	Cases	Slight (0 days)	Mild (1–7 days)	Moderate (8–28 days)	Severe (>28 days)	Total time-loss (days)
All Injuries	63	35	25	3	0	130
Acute injuries	17	7	9	1	0	43
Knee	4	1	2	1	0	22
Pelvis/lower back	3	2	1	0	0	2
Hip/groin	2	0	2	0	0	8
Head	1	0	1	0	0	1
Thigh	1	0	1	0	0	1
Elbow	1	1	0	0	0	0
Foot	1	1	0	0	0	0
Hand/fingers	1	1	0	0	0	0
Other/unspecified	3	1	2	0	0	9
Overuse injuries	46	28	16	2	0	87
Knee	10	8	2	0	0	9
Lower leg/Achilles	5	2	2	1	0	31
Foot	5	3	2	0	0	4
Hip/groin	5	5	0	0	0	0
Pelvis/lower back	3	2	1	0	0	5
Elbow	3	2	0	1	0	12
Ankle	3	1	2	0	0	3
Thigh	2	0	2	0	0	4
Upper arm	2	2	0	0	0	0
Head	1	0	1	0	0	4
Hand/fingers	1	0	1	0	0	2
Shoulder	1	0	1	0	0	1
Other/unspecified	5	3	2	0	0	12
Illnesses	45	7	35	3	0	173

Cases are categorised by days of time-loss (Slight 0, Mild 1–7, Moderate 8–28, Severe >28 days), following IOC consensus bands.

**Figure 3:**
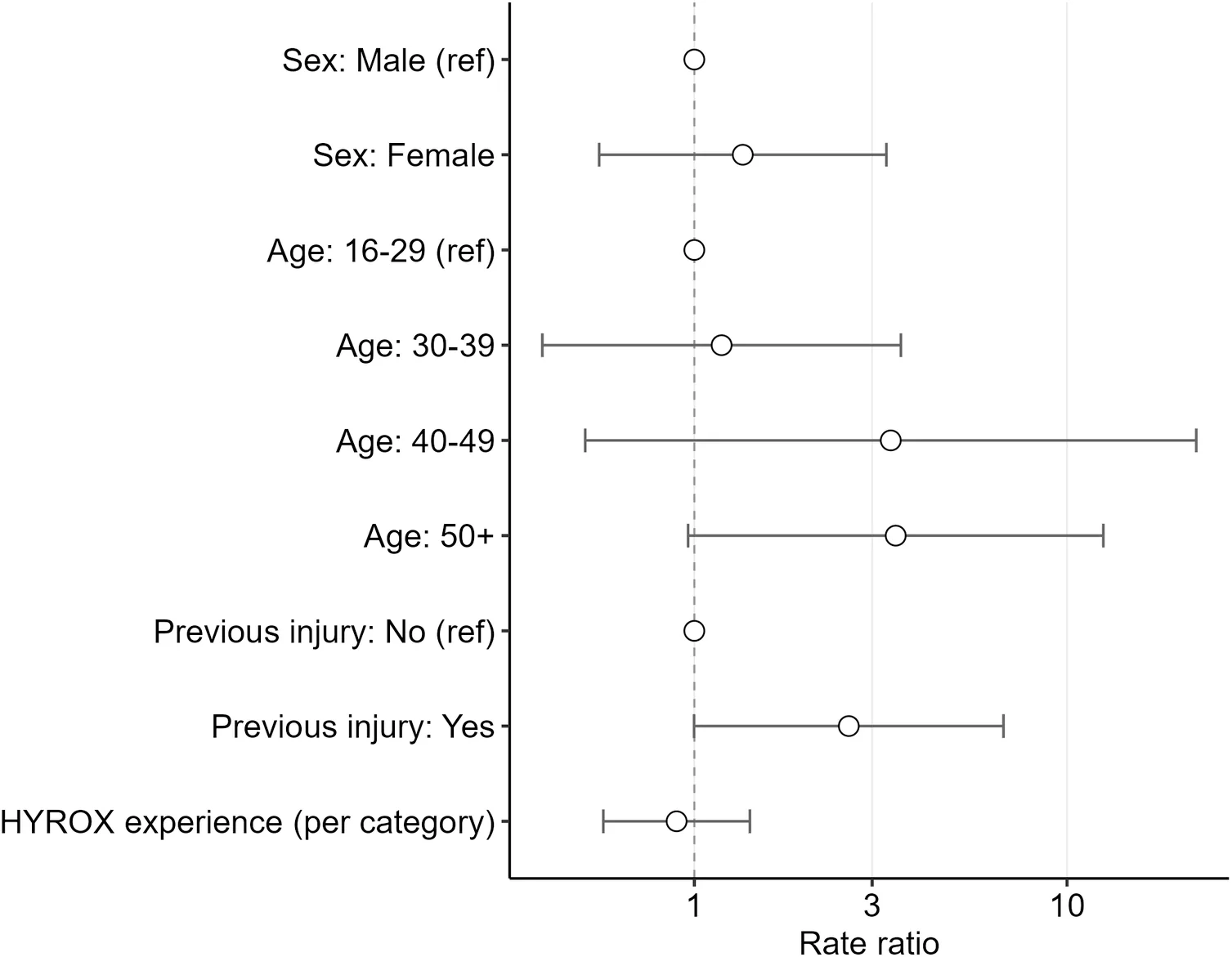
Forest plot of adjusted rate ratios for cumulative injury severity score. Reference categories shown at RR = 1. The *x*-axis is displayed on a logarithmic scale. Error bars represent 95% confidence intervals.

Rate ratios from the adjusted exploratory risk marker model are presented in Figure 3. A previous time-loss injury in the preceding 12 months carried the largest point estimate, at 2.60 for cumulative injury severity, but the confidence interval extended from 1.00 to 6.75 and is compatible with no association; the corresponding estimate in the HYROX-attributed sensitivity analysis was 2.95 (95% CI 1.06–8.22). Estimates for age, sex and HYROX experience were imprecise and are compatible with associations in either direction: female sex 1.35 (95% CI 0.56–3.28), age 30–39 years 1.18 (0.39–3.58), 40–49 years 3.37 (0.51–22.22), 50 years and above 3.47 (0.96–12.51), and HYROX experience 0.90 per category (0.57–1.41). Of the 89 participants, 84 contributed to this model; five participants provided no baseline data and were therefore excluded.

## 4 Discussion

This study presents the first prospective characterisation of health problem prevalence, anatomical distribution, and injury burden in HYROX athletes using weekly OSTRC-H2 surveillance. The injury profile was dominated by overuse injuries, which represented the substantive epidemiological problem in this population. Burden was concentrated in the knee and lower leg/Achilles, a distribution largely, though not entirely, typical of running populations. Training was continued despite symptoms in around half of problem-weeks, so a time-loss definition would have missed a substantial proportion of the health problems recorded in this population. In the exploratory risk marker analysis, previous injury was the marker most strongly associated with cumulative injury severity.

The pooled weekly prevalence of any health problem was 30%, and of substantial problems 18%. Both fall within the range reported for other populations monitored with OSTRC-H2 instruments. In the cohort used to develop the questionnaire (Norwegian cross-country skiers, cyclists, floorball players, handball players, and volleyball players) a mean weekly prevalence of 36% for any health problem and 15% for substantial problems was reported (26). A higher prevalence, at 43% for all problems and 25% for substantial problems, was reported in 320 youth elite athletes monitored across multiple sports (27). At the lower end, prevalences of 13% and 9% for all problems and substantial problems, respectively, were reported in a 45-week study of elite male handball players (28). Figures close to those observed in the current study were reported for women's Premier League footballers (32% any, 22% substantial) (29) and top-level football referees (34% any, 20% substantial) (30). Across these cohorts, HYROX appears comparable with regards to health problem prevalence. The fact that a sport involving high-intensity endurance running combined with repeated high-force efforts did not produce a substantially higher health problem load is noteworthy. However, the differing cohort ages, physiological demands, and study lengths mean these comparisons should be interpreted with caution.

Comparison of the injury incidence rates with the wider literature is complicated by differences in injury definitions. The all-complaint incidence of 14.4 per 1,000 h (95% CI 11.1–18.4) in the current study was generated by the OSTRC-H2 any-complaint definition, which counts the full symptomatic spectrum irrespective of time-loss or medical attention. The HYROX-attributed equivalent was 9.6 per 1,000 h (95% CI 6.9–13.0). An extreme conditioning program training meta-analytic estimate of 3.2 per 1,000 h (95% CI 2.06–4.34) was, by contrast, built on a strict three-part definition requiring at least three days of withdrawal, at least three days of modified training, or medical attention (8). A gap of this magnitude is therefore expected before any real difference in risk is invoked, since broader surveillance will inevitably detect substantially more problems than time-loss methods: roughly threefold in one direct comparison (13), and just over twofold between the all-complaint and time-loss rates within the present study. On a matched time-loss definition, the HYROX-attributed time-loss incidence in the present study was 4.3 per 1,000 h (95% CI 2.6–6.8), which is broadly comparable to the extreme conditioning program training estimate of 3.2 per 1,000 h (8), with substantially overlapping confidence intervals.

The running and resistance training comparators are weaker still. A pooled running injury incidence of 7.7 per 1,000 h (95% CI 6.9–8.7) (6) was assembled from studies with heterogeneous injury definitions, mostly consequence-based but with inconsistent thresholds, so the figure sits at an indeterminate point between the time-loss and all-complaint rates reported here and cannot be cleanly compared against either. The resistance training literature is similarly heterogeneous with respect to injury definitions. Serafim et al. (7) report rates of 4.2 per 1,000 h for HIFT-style training, 3.2 for weightlifting and 4.0 for powerlifting, but as narrative averages rather than pooled estimates, and on definitions that are not time-loss matched to the present study. On the evidence currently available, HYROX does not appear to carry a higher injury risk than the activities it combines. That said, a fatigue-mediated interaction between concurrent running and resistance loading, one that could raise risk beyond the sum of the parts, remains biologically plausible (31) and untested here. Larger cohorts, monitored under harmonised injury definitions, are needed before such comparisons can be settled.

The burden matrix was dominated by the knee and lower leg/Achilles, both of which are cleanly mapped onto the running injury literature, in which patellofemoral pain, iliotibial band syndrome, patellar tendinopathy and Achilles or calf complaints are common presentations (31, 32). Given that running was the single most frequently reported injury mechanism (43%), this is unsurprising. To that extent, a recognisably running-shaped lower-limb risk is carried by HYROX preparation. A disproportionate share of injury burden was accounted for by the elbow despite its being an infrequent site of injury. Four elbow episodes were recorded, around 6% of the total, but two of these persisted across multiple weeks and accumulated high cumulative severity, one being reported in every week of the surveillance period; together these two episodes account for 87% of the elbow's cumulative severity, and the elbow's third place in the burden ranking is lost if either is removed. The pattern is consistent with the repetitive gripping and pulling demands of several of the functional stations, such as the sled pull, farmers carry, SkiErg and rowing, but it rests on two participants and is offered as a hypothesis for testing in larger cohorts rather than as an established feature of the sport. The same caution applies to the near-absence of shoulder injuries, a single episode being recorded. The shoulder is the most frequently injured region across resistance training modalities generally, and individual extreme conditioning programme cohorts have located between roughly 15% and 46% of injuries there, driven largely by overhead barbell and gymnastic work (7, 33). No overhead lifting is contained in the HYROX station set, and the upper-limb load imposed is distal and pulling-dominant rather than proximal and pressing-dominant, so a distal upper-limb rather than shoulder-dominant profile is plausible. Whether this constitutes a genuine injury signature distinguishing HYROX from other extreme conditioning programme training cannot be determined from four and one episodes respectively. one episodes, respectively.Half of the health problems recorded did not involve time-loss, and because substantial problems were more prevalent than time-loss problems (18% against 15%), a subset of the non-time-loss complaints reduced training volume or performance without ever costing a full day. This is precisely the gap that OSTRC-style surveillance was designed to capture (11, 12, 15): the recording of overuse and non-time-loss complaints that conventional time-loss definitions omit. The operational consequence is that the true injury/illness burden will be systematically understated by any HYROX surveillance built on missed training days alone, and symptom-based tools are needed by practitioners if sound load-management decisions are to be made.

The exploratory risk marker analysis included 84 participants and was underpowered for stable estimation of most plausible effect sizes. Covariates were selected *a priori* on theoretical grounds rather than by data-driven search, following the cautions raised by Bahr and Holme (34) and Shrier and Platt (35) about model specification in injury risk-factor research. The aim was to flag candidate signals, not to estimate causal effects. Previous time-loss injury in the preceding 12 months was the marker most strongly associated with cumulative injury severity. The rate ratio was 2.60 (95% CI 1.00–6.75, *p* = 0.0504) in the primary all-injury model and 2.95 (95% CI 1.06–8.22, *p* = 0.039) in the HYROX-attributed sensitivity analysis, indicating roughly a two- to threefold higher cumulative severity. The direction and approximate magnitude are consistent with what is arguably the most replicated finding in sports injury epidemiology: injury risk is associated with prior injury, particularly with time-loss, across virtually every sport studied (21, 36).

The mechanism behind this association is not resolved by these data, and two accounts are worth distinguishing. Under a causal account, a previous injury directly raises the risk of a subsequent one through mechanisms such as residual tissue deficit, lingering neuromuscular control impairment, and altered movement strategies acquired during return to sport (37). Under a non-causal marker account, the previous injury does not itself raise subsequent risk but instead serves as a marker for traits that predispose to injury in the first place, whether genetic, behavioural such as risk-taking, or other injury-prone characteristics relating to training or psychology. On this view the previous injury and the subsequent injury share a common cause, and the observed association reflects confounding rather than a causal effect (35). The present design, a short observational cohort study with baseline covariates and no measurement of these upstream traits, cannot adjudicate between the two, and previous injury is best treated here as a risk marker rather than an established risk factor. The practical implication is, however, robust to which account holds: athletes returning from a recent injury warrant graduated reintroduction and closer monitoring.

No clear association was shown for the remaining markers, but the estimates are too imprecise for the absence of an association to be inferred. The interval for female sex, 0.56–3.28, excludes only differences larger than around threefold in either direction. That for the 40–49 years group spans 0.51–22.22, a range across which no useful inference is possible, and that for the 50 years and above group, 0.96–12.51, is compatible with anything from a slight protective association to a twelvefold increase. Point estimates for the older age groups were elevated relative to the 16–29 years reference, with a possible gradient across categories, and if age does modify HYROX injury risk a much larger cohort will be needed for its detection; the healthy-survivor dynamic, by which less injury-resilient masters athletes are selectively removed from training through dropout, should be anticipated in any such study (38). Several practical messages follow from the burden profile, and these are summarised in Table 4. The lower limb accounted for most of the burden recorded here, with the knee ranking first on both frequency and burden and the lower leg and Achilles second on burden despite only five episodes. Running was the mechanism most often reported for lower-limb episodes, although a mechanism was recorded for fewer than half of all episodes and the modality-specific rates in Supplementary Table S2 are correspondingly imprecise. Two features of the profile relate to how surveillance should be conducted rather than on what should be prevented. Nine of the fourteen knee episodes involved no time loss, and only four days were lost across six foot episodes, so an injury definition reliant on missed training days would have recorded little of the burden carried by either region. For the hip, groin, pelvis, and lower back no specific target is indicated as the reported mechanisms were mixed and the proportion of sudden-onset episodes was higher. It is emphasised that these are candidate targets derived from a descriptive profile in a single cohort of predominantly novice athletes, that several rest on small numbers of episodes, and that none has been evaluated.

**Table 4:** Highest-burden injury locations, reported mechanisms, and candidate prevention targets.

Body region	Episodes, *n* (%)	Gradual-onset, %	Days lost	Reported mechanisms	Candidate targets for prevention (untested)
Knee	14 (22)	71	31	Running 5, strength 2, station 2 (9 of 14 recorded)	Progression of running volume; routine enquiry about knee symptoms that do not cause time loss, since 9 of 14 episodes involved none
Lower leg/Achilles	5 (8)	100	31	Running 2 (2 of 5 recorded)	Calf and Achilles loading capacity; progression of running volume. Highest mean cumulative severity of any region despite few episodes
Elbow	4 (6)	75	12	Station 2, strength 1 (3 of 4 recorded)	Grip and elbow loading tolerance for the pulling and carrying stations. Rests on four episodes, two of which account for 87% of the region's cumulative severity
Foot	6 (10)	83	4	Running 2, strength 1 (3 of 6 recorded)	Progression of running volume. Burden accrues through persistence rather than time loss; only 4 days were lost across 6 episodes
Hip/groin	7 (11)	71	8	Running 2, station 1, strength 1 (4 of 7 recorded)	No specific target indicated. Mechanisms were mixed and no single exposure predominated
Pelvis/lower back	6 (10)	50	7	Strength 2, station 1 (3 of 6 recorded)	No specific target indicated. Half of these episodes were of sudden onset, and the reported mechanisms do not isolate a single activity

Regions are ordered by injury burden (incidence multiplied by mean cumulative severity) and together account for 42 of the 63 injury episodes recorded. Percentages of episodes are of all 63 episodes. Gradual-onset denotes injuries classified as overuse rather than sudden onset. Reported mechanisms are counts of episodes attributed by the participant to each activity, with the number of episodes for which any mechanism was recorded given in parentheses; mechanism was not recorded for 33 of 63 episodes overall, and was recorded less often for injuries that participants did not attribute to HYROX training. No prevention strategy was evaluated in this study, and the final column lists targets that the burden profile makes plausible rather than interventions shown to be effective in this population. All entries are drawn from a single 12-week cohort of 89 predominantly novice athletes and several rest on small numbers of episodes.

### 4.1 Limitations

What can be concluded from these data is bounded by several limitations. The most consequential is sample size. With 84 participants contributing to the risk marker analysis, the study is underpowered for stable estimation of all but large effects, as indicated by the wide confidence intervals observed throughout the regression analysis. All injuries, illnesses and exposures were participant-reported. The OSTRC-H2 shows high agreement with clinician records (82% for injury and 94% for illness) (39), but the 62% mean weekly response rate leaves health problems arising in unreported weeks unobserved, so true incidence is plausibly higher than recorded. Two features limit the consequences; most missing data arose from withdrawal rather than intermittent non-response, and pooled weekly prevalence was almost unchanged when gaps between two returned questionnaires were imputed. Exposure accrued only over returned weeks, so the incidence denominator was not inflated by non-response. Set against this, response in the following week was slightly less likely after a week in which a problem had been reported, and participants who completed fewer weeks reported more problems while they remained in the study, both of which point towards modest under-ascertainment. A prize draw conditional on 80% completion may additionally have selected for engaged participants. Self-report also carries some misclassification risk in the body-region and mechanism classifications.

The cohort may not be representative of the global HYROX population. Recruitment was multi-modal but weighted towards UK-based athletes with enough digital engagement to complete a weekly online questionnaire, which limits generalisability to other regions and to less-engaged participants. The temporal reach of the findings is constrained by the 12-week observation window. Chronic conditions requiring longer cumulative loading to manifest may be underrepresented, and seasonal variation in load and risk cannot be captured. All covariates were measured at baseline, so time-varying contributors to weekly risk, acute load spikes, sleep, and life stress among them, were not modelled, despite being plausible drivers of week-to-week injury occurrence. Total exposure was recorded as a weekly sum by modality but with no information on its distribution within the week, on session intensity, or on week-to-week change. Weekly exposure varied widely, both between participants and within them across the surveillance period, and cumulative exposure entered the model only as an offset. Time-varying load was therefore not modelled. Beyond the limited number of episodes available, weekly load and weekly health status are contemporaneously confounded, because training is reduced in the week in which a problem is reported, and injury onset is interval-censored to the reporting week; a lagged or time-varying model built on these data would be dominated by the effect of injury on load rather than the reverse.

Eligibility required only three weeks of HYROX-specific training, a threshold chosen so that the sample would reflect the population the event actually attracts, which is dominated by recent entrants, rather than a more experienced subgroup; the consequence is that the estimates reported here describe a predominantly novice cohort. Median reported training exposure was 6.0 h per week (IQR 3.5–9.0), 62% had under a year of HYROX experience, and although 46% had competed in the preceding 12 months, competition accounted for 2.5% of all recorded exposure and fewer than half of participants logged any competition hours during the surveillance period. The findings should therefore not be extrapolated to elite or long-tenured competitors, whose training volumes, intensities and competition density may differ substantially from those observed here. The direction of the resulting bias cannot be determined. Inexperience may carry elevated risk, in which case the rates reported here overstate those of the wider HYROX population; equally, experienced athletes accumulate greater exposure at higher intensities and compete more often, which would act in the opposite direction. Training age and competitive standard should be stratified on in future work. Finally, the comparisons drawn with other sports must be read with definitional caution. The comparator incidence rates are based on differing injury definitions, and only the matched time-loss comparison with extreme conditioning program training is on a common footing. The prevalence comparisons are sounder, because the same OSTRC instrument was used by the comparator studies, but even these differ in cohort age, sport demands, and monitoring duration.

These limitations notwithstanding, a more rigorous epidemiological baseline for HYROX than has previously existed is provided by the study's prospective design, weekly monitoring with an IOC-aligned validated instrument, modality-disaggregated exposure measurement, burden quantification rather than incidence alone, and *a priori* theory-driven covariate selection. Four priorities follow from the limitations of this study. Multicentre international cohorts are needed, recruited across competitive standards and with exposure logged by modality, so that rates can be attributed to the activities that generate them rather than to training as a whole. Follow-up should extend across a full competitive season and include the taper and race periods. Objective monitoring of external and internal load, collected at a resolution that permits time-varying analysis, would allow the relationship between load and health status to be examined without the reverse causality that constrains weekly self-report. And sex-stratified analyses in samples large enough to support them are required, since the present cohort was underpowered for any stratified comparison. Sleep, recovery and psychological variables, and biomechanical assessment of the loaded station movements, are further plausible avenues, although these would be better pursued once the descriptive baseline has been established across a broader population.

### 4.2 Conclusion

This study provides the first prospective epidemiological description of health problems in HYROX athletes. The injury burden is dominated by overuse injuries, with a mean weekly health-problem prevalence comparable to other athletic populations monitored with the same instrument. The injury profile appears hybrid: lower-limb burden is concentrated in the knee and lower leg, consistent with the sport's substantial running load, alongside a possible elbow burden, based on four episodes, that may arise from the pulling, carrying and gripping demands of the functional stations. When injury rates are compared on a matched time-loss definition, HYROX appears no more hazardous than other HIFT-style training. A central practical finding is that around half the health problems did not involve time-loss yet training was still affected, so the true burden will be substantially understated by surveillance and load management built on missed-training days alone. A previous time-loss injury was flagged by the exploratory analysis as the leading candidate risk marker associated with cumulative injury severity, consistent with the wider sports injury literature, while no clear association was shown for sex, age, or HYROX experience in this sample. Confirmation in adequately powered, multi-site prospective cohorts with time-varying exposure measurement is required before these signals can guide practice. In the meantime, the burden profile points to the lower limb as the site at which prevention effort may have most impact, although no intervention was evaluated here and the effectiveness of running-load management or of lower-limb tendon-capacity work in this population remains untested. As participation in HYROX continues to grow, an initial evidence base on which prevention strategies and further research can be built is offered by these findings.

## Data Availability

The dataset presented in this article is not readily available because participants did not consent to public deposition of their individual weekly health and training records, and the granularity of these data (weekly injury reports, body region, and training exposure over twelve weeks) could permit reidentification within a small and geographically concentrated cohort. De-identified data may be made available to researchers for projects that receive a favourable ethical opinion from the University of Bath, in accordance with participant consent. Requests to access the datasets should be directed to Sean Williams, S.Williams@bath.ac.uk. The analysis code (R) is openly available under the MIT licence in the University of Bath Research Data Archive: https://doi.org/10.15125/BATH-01699.

## References

[1] HYROX. Hyrox Rulebook Single: Season 25/26. (2025). Available from: Available online at: https://maintain.hyrox.com/rulebooks/HYROX_RulebookSingles_EN.pdf (Accessed May 20, 2026).

[2] Fernández-Navarrete I Ruiz-Alias SA Gómez-García M Riquelme-Sebastián L García-Pinillos F. Hyrox©: a descriptive analysis of participation trends among over 275,000 athletes in the new fitness modality. Res Q Exerc Sport. (2026) 97(2):266–72. 10.1080/02701367.2025.2584226 41380137

[3] HYROX Sports Science Advisory Council. The Hyrox Sports Science Report 2025. (2025).

[4] Villarroel López P Agudo-Ortega A Santos-García DJ. Characterization of the profile of Hyrox© athletes. Appl Sci. (2025) 15(21):11693. 10.3390/app152111693

[5] Coughlin SS. Recall bias in epidemiologic studies. J Clin Epidemiol. (1990) 43(1):87–91. 10.1016/0895-4356(90)90060-3 2319285

[6] Videbæk S Bueno AM Nielsen RO Rasmussen S. Incidence of running-related injuries per 1000 H of running in different types of runners: a systematic review and meta-analysis. Sports Med. (2015) 45(7):1017–26. 10.1007/s40279-015-0333-8 PMC4473093 25951917

[7] Serafim TT de Oliveira ES Maffulli N Migliorini F Okubo R. Which resistance training is safest to practice? A systematic review. J Orthop Surg Res. (2023) 18(1):296. 10.1186/s13018-023-03781-x 37046275 PMC10099898

[8] Hülsmann M Reinecke K Barthel T Reinsberger C. Musculoskeletal injuries in Crossfit®: a systematic review and meta-analysis of injury rates and locations. Dtsch Z Sportmed. (2021) 72(2):351–8. 10.5960/dzsm.2021.504

[9] Brandt T Ebel C Lebahn C Schmidt A. Acute physiological responses and performance determinants in Hyrox(©) - a new running-focused high intensity functional fitness trend. Front Physiol. (2025) 16:1519240. 10.3389/fphys.2025.1519240 40230601 PMC11994925

[10] Davids CJ. A performance analysis of Hyrox: a review of the physiologic, mechanical, and technical demands. Strength Cond J. (2022) 48(1):88–100. 10.1519/SSC.0000000000000913

[11] Bahr R. No Injuries, but Plenty of Pain? On the methodology for recording overuse symptoms in sports. Br J Sports Med. (2009) 43(13):966–72. 10.1136/bjsm.2009.066936 19945978

[12] Clarsen B Myklebust G Bahr R. Development and validation of a new method for the registration of overuse injuries in sports injury epidemiology: the Oslo Sports Trauma Research Centre (OSTRC) overuse injury questionnaire. Br J Sports Med. (2013) 47(8):495–502. 10.1136/bjsports-2012-091524 23038786

[13] Mashimo S Hogan T Nishida S Watanabe Y Matsuki Y Suhara H. Influence of surveillance methods in the detection of sports injuries and illnesses. Int J Sports Phys Ther. (2022) 17(6):1119–27. 10.26603/001c.37852 36237647 PMC9528695

[14] Lakens D. Sample size justification. Collabra Psychol. (2022) 8(1):33267. 10.1525/collabra.33267.

[15] Clarsen B Bahr R Myklebust G Andersson SH Docking SI Drew M. Improved reporting of overuse injuries and health problems in sport: an update of the Oslo sport trauma research center questionnaires. Br J Sports Med. (2020) 54(7):390–6. 10.1136/bjsports-2019-101337 32060142

[16] Venables WN Ripley BD. Modern Applied Statistics with S. New York, NY: Springer Science & Business Media (2013).

[17] Zeileis A Köll S Graham N. Various Versatile variances: an object-oriented implementation of clustered covariances in R. J Stat Softw. (2020) 95:1–36. 10.18637/jss.v095.i01

[18] Canty A Ripley B. boot: Bootstrap Functions. (2025). doi: 10.32614/CRAN.package.boot. R package version 1.3-32. Available online at: https://CRAN.R-project.org/package=boot.

[19] Bahr R Clarsen B Derman W Dvorak J Emery CA Finch CF. International Olympic Committee consensus statement: methods for recording and reporting of epidemiological data on injury and illness in sport 2020 [including STROBE extension for sport injury and illness surveillance (STROBE-SIIS)]. Br J Sports Med. (2020) 54(7):372–89. 10.1136/bjsports-2019-101969 32071062 PMC7146946

[20] Bahr R Clarsen B Ekstrand J. Why we should focus on the burden of injuries and illnesses, not just their incidence. Br J Sports Med. (2018) 52(16):1018–21. 10.1136/bjsports-2017-098160 29021247

[21] Fulton J Wright K Kelly M Zebrosky B Zanis M Drvol C. Injury risk is altered by previous injury: a systematic review of the literature and presentation of causative neuromuscular factors. Int J Sports Phys Ther. (2014) 9(5):583. 25328821 PMC4196323

[22] Hollander K Rahlf AL Wilke J Edler C Steib S Junge A. Sex-Specific differences in running injuries: a systematic review with meta-analysis and meta-regression. Sports Med. (2021) 51(5):1011. 10.1007/s40279-020-01412-7 33433864 PMC8053184

[23] Moran S Booker H Staines J Williams S. Rates and risk factors of injury in CrossFittm: a prospective cohort study. J Sports Med Phys Fitness. (2017) 57(9):1147–53. 10.23736/s0022-4707.16.06827-4 28085123

[24] Sonesson S Lindblom H Hägglund M. Higher age and present injury at the start of the season are risk factors for in-season injury in amateur male and female football players—a prospective cohort study. Knee Surg Sports Traumatol Arthrosc. (2023) 31(10):4618–30. 10.1007/s00167-023-07517-6 37542529 PMC10471640

[25] Feito Y Burrows EK Tabb LP. A 4-year analysis of the incidence of injuries among crossfit-trained participants. Orthop J Sports Med. (2018) 6(10):2325967118803100. 10.1177/2325967118803100 30370310 PMC6201188

[26] Clarsen B Rønsen O Myklebust G Flørenes TW Bahr R. The Oslo sports trauma research center questionnaire on health problems: a new approach to prospective monitoring of illness and injury in elite athletes. Br J Sports Med. (2014) 48(9):754–60. 10.1136/bjsports-2012-092087 23429267

[27] Moseid CH Myklebust G Fagerland MW Clarsen B Bahr R. The prevalence and severity of health problems in youth elite sports: a 6-month prospective cohort study of 320 athletes. Scand J Med Sci Sports. (2018) 28(4):1412–23. 10.1111/sms.13047 29281145

[28] Drole K Busch A Paravlic A Doupona M Prevalence SK. Incidence and burden of health problems across playing positions in elite male handball players: a 45-week prospective cohort study. BMJ Open Sport Exerc Med. (2025) 11(2):e002460. 10.1136/bmjsem-2025-002460 40195973 PMC11973782

[29] Amundsen R Thorarinsdottir S Clarsen B Andersen TE Møller M Bahr R. #Readytoplay: health problems in women’s football-a two-season prospective cohort study in the Norwegian premier league. Br J Sports Med. (2023) 58:4–10. 10.1136/bjsports-2023-107141 37968072

[30] Moen C Andersen TE Clarsen B Madsen-Kaarød G Dalen-Lorentsen T. Prevalence and burden of health problems in top-level football referees. Sci Med Footb. (2023) 7(2):131–8. 10.1080/24733938.2022.2055782 35430956

[31] Lopes AD Hespanhol LC Jr Yeung SS Costa LOP. What are the main running-related musculoskeletal injuries? A systematic review. Sports Med. (2012) 42(10):891–905. 10.1007/bf03262301 22827721 PMC4269925

[32] van Gent RN Siem D van Middelkoop M van Os AG Bierma-Zeinstra SM Koes BW. Incidence and determinants of lower extremity running injuries in long distance runners: a systematic review. Br J Sports Med. (2007) 41(8):469–80. 10.1136/bjsm.2006.033548 17473005 PMC2465455

[33] Klimek C Ashbeck C Brook AJ Durall C. Are injuries more common with crossfit training than other forms of exercise? J Sport Rehabil. (2018) 27(3):295–9. 10.1123/jsr.2016-0040 28253059

[34] Bahr R Holme I. Risk factors for sports injuries–a methodological approach. Br J Sports Med. (2003) 37(5):384–92. 10.1136/bjsm.37.5.384 14514527 PMC1751357

[35] Shrier I Platt RW. Reducing bias through directed acyclic graphs. BMC Med Res Methodol. (2008) 8:70. 10.1186/1471-2288-8-70 18973665 PMC2601045

[36] Toohey LA Drew MK Cook JL Finch CF Gaida JE. Is subsequent lower limb injury associated with previous injury? A systematic review and meta-analysis. Br J Sports Med. (2017) 51(23):1670–8. 10.1136/bjsports-2017-097500 28784622

[37] Hewett TE Di Stasi SL Myer GD. Current concepts for injury prevention in athletes after anterior cruciate ligament reconstruction. Am J Sports Med. (2013) 41(1):216–24. 10.1177/0363546512459638 23041233 PMC3592333

[38] Ganse B Degens H Drey M Korhonen M McPhee J Müller K. Impact of age, performance and athletic event on injury rates in master athletics–first results from an ongoing prospective study. J Musculoskelet Neuronal Interact. (2014) 14(2):148–54. 24879018

[39] Fitch AS Mara J Hetherington M Mahony K Drew MK Waddington G. The validity and engagement of the ostrc-H2 questionnaire as a surveillance tool to detect health problems in a high-performance Australian youth diving cohort: an observational study. Health Sci Rep. (2025) 8(4):e70654. 10.1002/hsr2.70654 40256150 PMC12007178

