# Supplementary material for: Prevalence, burden and risk markers of health problems in HYROX athletes: a 12-week prospective cohort study
Source: Front Sports Act Living. 2026 Sep 9;8:1937574. doi: 10.3389/fspor.2026.1937574 (PMC13597903; doi:10.3389/fspor.2026.1937574)
Supplement: Supplementary file 1 [file Table1.docx]

**Supplementary material**

*Prevalence, Burden and Risk Markers of Health Problems in HYROX Athletes: A 12-Week Prospective Cohort Study*

**Supplementary Table S1. Sensitivity of injury incidence to the construction of the exposure denominator and to the episode-reconstruction rules.**

| **Specification** | **Episodes** | **Exposure, h** | **All injuries per 1000 h (95% CI)** | **Time-loss injuries per 1000 h (95% CI)** |
| --- | --- | --- | --- | --- |
| Primary analysis, as reported | 63 | 4376 | 14.4 (11.1 to 18.4) | 6.4 (4.3 to 9.2) |
| Denominator from weekly modality items | 63 | 4283 | 14.7 (11.3 to 18.8) | 6.5 (4.3 to 9.4) |
| Recovery work excluded from denominator | 63 | 3749 | 16.8 (12.9 to 21.5) | 7.5 (5.0 to 10.8) |
| Gap of >2 weeks closes an episode | 63 | 4376 | 14.4 (11.1 to 18.4) | 6.4 (4.3 to 9.2) |
| Any missing week closes an episode | 63 | 4376 | 14.4 (11.1 to 18.4) | 6.4 (4.3 to 9.2) |
| Unattached injury weeks counted as incident | 66 | 4376 | 15.1 (11.7 to 19.2) | 6.4 (4.3 to 9.2) |

Confidence intervals are exact Poisson intervals. Recovery work comprises prehabilitation and stretching and accounted for 12.5% of total reported hours. Episode counts are determined by the number of recorded injury onsets and are therefore unchanged by rules governing when an episode closes; those rules affect only the aggregation of severity and time-loss days within episodes, which did not differ across the specifications examined. Unattached injury weeks are injury weeks for which no onset had been reported earlier in that participant's record; four such weeks occurred, forming three runs in three participants, none involving time loss.

**Supplementary Table S2. Exploratory modality-specific injury incidence.**

| **Mechanism** | **Episodes** | **Share of recorded mechanisms** | **Modality exposure, h** | **Share of total exposure** | **Injuries per 1000 h of that modality (95% CI)** |
| --- | --- | --- | --- | --- | --- |
| Running | 13 | 43% | 1144 | 26.7% | 11.4 (6.1 to 19.4) |
| HYROX station work | 9 | 30% | 1179 | 27.5% | 7.6 (3.5 to 14.5) |
| Strength training | 8 | 27% | 1321 | 30.8% | 6.1 (2.6 to 11.9) |
| Not recorded | 33 | — | — | — | — |

Numerators are injury episodes attributed by the participant to each mechanism; denominators are the corresponding modality-specific exposure hours summed across the cohort. A mechanism was recorded for 30 of 63 episodes (48%), and less often for injuries not attributed to HYROX training (67% missing) than for those that were (44% missing). Because of the extent and the differential nature of this missingness, and the width of the confidence intervals, these rates should not be interpreted as a ranking of modality-specific risk.

**Supplementary Table S3. Baseline characteristics of participants above and below the median number of completed weekly questionnaires.**

| **Characteristic** | **≥9 weeks completed (n = 45)** | **<9 weeks completed (n = 44)** | **SMD** |
| --- | --- | --- | --- |
| Age group (1–9 ordinal scale) | 4.22 | 3.72 | 0.22 |
| Female | 67% | 59% | 0.16 |
| HYROX experience (1–5 ordinal scale) | 2.33 | 2.23 | 0.09 |
| Previous time-loss injury | 44% | 43% | 0.03 |
| Baseline total training, h/week | 7.78 | 7.79 | −0.01 |
| Baseline HYROX training, h/week | 2.98 | 3.38 | −0.21 |
| Competed in previous 12 months | 49% | 43% | 0.11 |

Participants were divided at the median of nine completed weekly questionnaires. Values are means for continuous variables and percentages for binary variables. SMD, standardised mean difference; absolute values below 0.25 were taken to indicate acceptable balance. Age group and HYROX experience are ordinal codes rather than years, so their means are reported on the coding scale.

**Supplementary Table S4. Pooled prevalence of health problems under alternative assumptions about non-response.**

| **Outcome** | **Returned weeks only** | **Interior-gap LOCF** | **Non-response assumed problem-free** |
| --- | --- | --- | --- |
| Any health problem | 29.9% | 29.9% | 18.4% |
| Substantial health problem | 17.9% | 18.1% | 11.0% |
| Time-loss health problem | 15.0% | 15.3% | 9.3% |

Values are pooled across returned participant-weeks. Interior-gap LOCF carries the last observation forward across gaps that lie between two returned questionnaires and does not extrapolate beyond a participant's final response, so it addresses intermittent non-response but not withdrawal. The final column assumes that every week without a returned questionnaire was free of health problems and therefore represents a lower bound. LOCF, last observation carried forward.
